# Wrist speed feedback improves elbow compensation and reaching accuracy for myoelectric transradial prosthesis users in hybrid virtual reaching task

**DOI:** 10.1186/s12984-023-01138-3

**Published:** 2023-01-19

**Authors:** Eric J. Earley, Reva E. Johnson, Jonathon W. Sensinger, Levi J. Hargrove

**Affiliations:** 1 Center for Bionic Medicine, Shirley Ryan AbilityLab, Chicago, IL USA; 2 Department of Biomedical Engineering, Northwestern University, Chicago, IL USA; 3 Center for Bionics and Pain Research, Mölndal, Sweden; 4 Department of Electrical Engineering, Chalmers University of Technology, Gothenburg, Sweden; 5 Department of Mechanical Engineering and Bioengineering, Valparaiso University, Valparaiso, IN USA; 6 Institute of Biomedical Engineering, University of New Brunswick, Fredericton, NB Canada; 7 Department of Electrical and Computer Engineering, University of New Brunswick, Fredericton, NB Canada; 8 Department of Physical Medicine and Rehabilitation, Northwestern University, Chicago, IL USA

**Keywords:** Sensory feedback, Myoelectric prosthesis, Center-out reaching, Compensatory movement, Motor adaptation, Motor learning

## Abstract

**Background:**

Myoelectric prostheses are a popular choice for restoring motor capability following the loss of a limb, but they do not provide direct feedback to the user about the movements of the device—in other words, kinesthesia. The outcomes of studies providing artificial sensory feedback are often influenced by the availability of incidental feedback. When subjects are blindfolded and disconnected from the prosthesis, artificial sensory feedback consistently improves control; however, when subjects wear a prosthesis and can see the task, benefits often deteriorate or become inconsistent. We theorize that providing artificial sensory feedback about prosthesis speed, which cannot be precisely estimated via vision, will improve the learning and control of a myoelectric prosthesis.

**Methods:**

In this study, we test a joint-speed feedback system with six transradial amputee subjects to evaluate how it affects myoelectric control and adaptation behavior during a virtual reaching task.

**Results:**

Our results showed that joint-speed feedback lowered reaching errors and compensatory movements during steady-state reaches. However, the same feedback provided no improvement when control was perturbed.

**Conclusions:**

These outcomes suggest that the benefit of joint speed feedback may be dependent on the complexity of the myoelectric control and the context of the task.

## Background

For individuals living with upper limb loss or difference, myoelectric prostheses have the potential to restore lost functionality and improve independence. Significant advancements have been made in myoelectric control methods, but sensory feedback is still a missing component from commercial prostheses. Sensory feedback is one of the most commonly requested features of state-of-the-art prostheses [1], and is critical to able-bodied limb control [2]. Consequently, artificial sensory feedback has received much attention over the past decade [3, 4]. Typically, this takes the form of sensory substitution feedback, where the information provided from missing sensory organs is communicated to the user via an alternative method such as vibrotactile [5–10] or auditory stimuli [11–13], or via direct nerve stimulation [14–16].

Despite this attention, artificial sensory feedback has not yet achieved commercial availability for prostheses, which may be related in part to the experimental conditions in which these systems are tested. Frequently, artificial feedback is tested with subjects blindfolded and not connected to the prosthesis. Although these studies consistently show the benefit of sensory feedback, they omit the incidental sources of feedback that prosthesis users rely on every day, such as vision, sound, and prosthesis vibration. This incidental feedback often serves the same purpose as the artificial feedback being tested (i.e. informing the user about the state of the prosthesis), and studies have shown this incidental feedback is sufficient for some tasks [17]. Therefore, when artificial feedback is tested *alongside* incidental feedback, results become inconsistent—some studies suggest discernable benefits of artificial feedback alongside incidental feedback, such as improved time to target prosthesis position [18], ability to perform object manipulation tasks [19], and coordination of grasping with the prosthesis [20], however the same and other studies also show no changes in other aspects of prosthesis use [9, 19, 21–24].

One theory explaining this discrepancy stems from the degree of precision of each feedback source. When we receive the same information from multiple sources, we merge them in accordance with their uncertainty: sources with less uncertainty are favored over those with greater uncertainty [25, 26]. Therefore, if incidental feedback (particularly vision) is more precise than the artificial feedback being tested, then the tested feedback may not meaningfully improve the users understanding of their prosthesis movements.

One candidate for sensory feedback which is not well estimated by incidental vision is kinesthesia. Prior work has suggested that limb speed, and in particular joint speed, has high visual uncertainty and can be supplemented with audio feedback to greatly reduce this uncertainty [27]. Knowledge of limb speed may aid in the formation of internal models of biological and prosthetic limb movements, and a previous study has indeed suggested that supplemental joint speed feedback may improve reaching accuracy during instances of perturbed myoelectric control [28]. However, the benefits of such feedback requires additional investigation within the context of impaired proprioception following amputation. Proprioceptive organs including muscle spindles and Golgi tendon organs are activated differently in an amputated limb than they are in intact limb; agonist–antagonist muscles pairs stimulate these organs during movement [29], but this pairing is generally absent from amputated limbs. Instead, standard surgical procedure for upper-limb amputations involves myodesis of the muscles to the end of the distal bone, preventing the normal passive stretching of antagonist muscles during movement and negatively affecting proprioception [30].

The purpose of this study was to investigate the effect of joint speed feedback on prosthesis control and adaptation to errors during reaching. Transradial amputee subjects controlled a virtual 1-DoF myoelectric limb and completed center-out reaching tasks under steady-state and perturbed dynamics conditions. We quantified control by measuring trial-by-trial adaptation to self-generated and perturbation-generated errors to learn how quickly myoelectric control users can update their understanding of the dynamics and adjust accordingly.

## Methods

### Subjects

Six subjects with transradial amputation participated in this study (Table 1), which was approved by the Northwestern University Institutional Review Board; all experiments were performed in accordance with relevant guidelines and regulations, and all subjects provided informed consent before starting the study.

**Table 1:** Transradial amputee subject demographics

Subject ID	Sex	Age	Side of amputation	Years since amputation	Cause of amputation	Home prosthesis	Familiarity with myoelectric control
TR1	M	71	R	32	Trauma	Passive	Familiar from participation in research studies
TR2	M	33	L	5	Trauma	Myoelectric, multiarticulate hand	Daily user of myoelectric pattern recognition, 5 years
TR3	M	28	R	10	Trauma	Body-powered	Familiar from participation in research studies
TR4	M	56	R	40	Trauma	Myoelectric, multiarticulate hand	Daily user of two-site myoelectric control, 5+ years
TR5	F	60	R	6	Cancer	Passive	Familiar from participation in research studies
TR6	M	65	L	6	Trauma	Body-powered	Previous myoelectric pattern recognition user

### Experimental setup

**Fig. 1:**
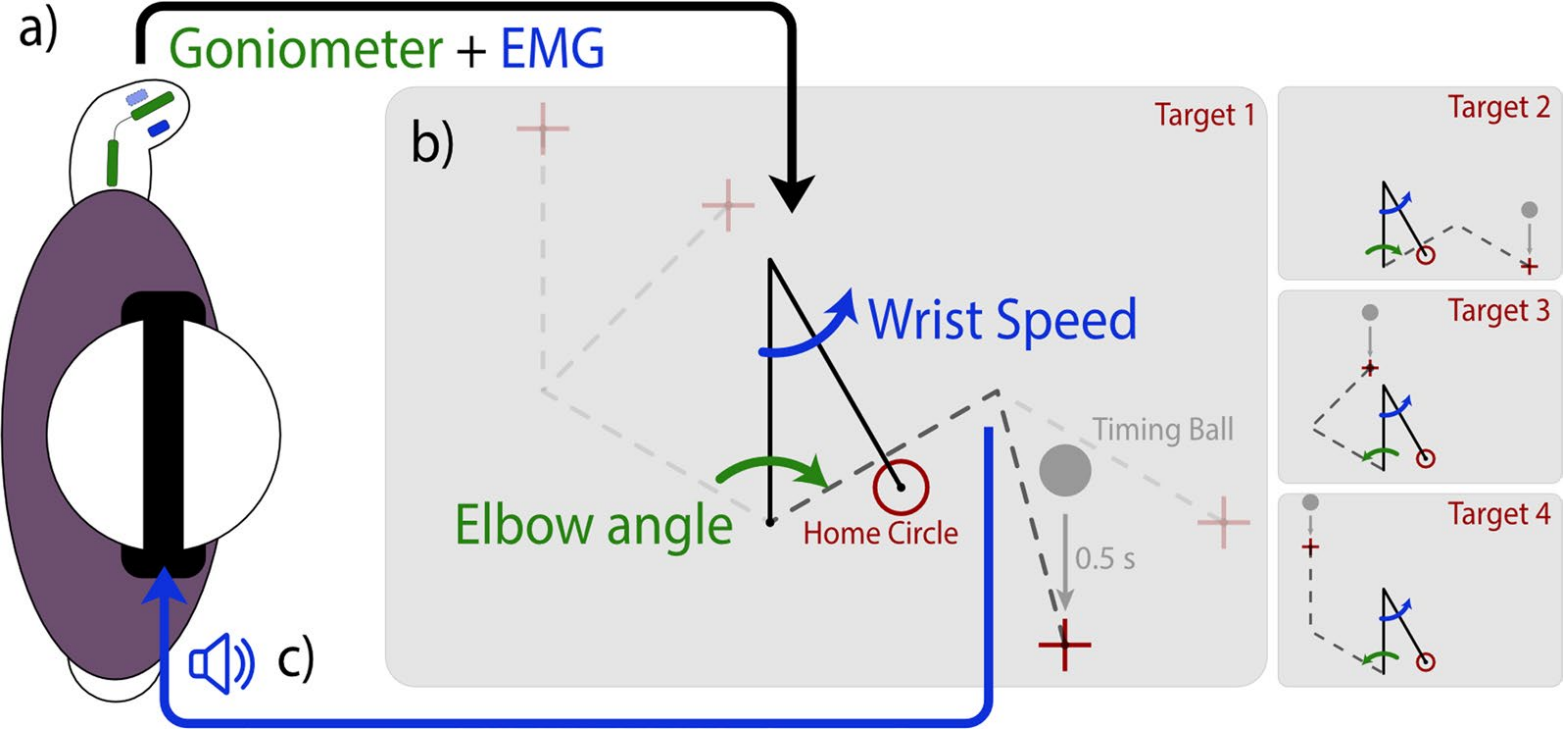
Center-out reaching experiment setup for a subject with left-side amputation. **a** Subject holds their arm in a relaxed posture at their side. Attached to the subject’s residual limb, a goniometer (green) measures elbow angle, and EMG sensors (blue) measure EMG amplitude. **b** Subjects perform center-out reaches with a virtual limb (black); goniometer angle controls the angle of the proximal link (or elbow, green), and the EMG amplitude controls the speed of the distal link (or wrist, blue). Subjects started with the limb endpoint in the home circle and one of four targets would appear. A grey ball would appear above the target; each target could only be reached with a single limb configuration (dashed grey, not shown on the screen). When the limb endpoint left the home circle, the ball began to drop, centering on the target after 0.5 s, signifying the end of the trial. The virtual task was mirrored for subjects with right-side amputation. **c** Wrist-driven distal link speed is used for frequency-modulated audio feedback, with higher speed corresponding to higher frequency. This audio feedback was played through headphones worn by the subject, providing wrist speed feedback

Subjects sat in front of a computer monitor displaying a virtual arm. A Biometrics twin-axis electrogoniometer was attached to the upper and lower arm to measure the elbow flexion angle. Goniometer signals were low-pass filtered at 5 Hz with a 2nd order Butterworth filter. Two Delsys Bagnoli electromyographic (EMG) sensors measured EMG signals from wrist flexor and extensor sites on the residual limb (Fig. 1a). The electrode placement was determined via voluntary muscle contraction and palpation (similar to the method used to place electrodes when controlling a myoelectric prosthesis), and the reference electrode was placed over the olecranon or on the clavicle. EMG signals were high-pass filtered at 0.1 Hz, positive-rectified, and low-pass filtered at 5 Hz using a 2nd order Butterworth filter. Data were acquired at 1000 Hz and downsampled to 100 Hz after filtering.

Subjects controlled a virtual two-link arm using the elbow goniometer to dictate proximal link position, and the wrist EMG sensors to dictate distal link velocity (Fig. 1b). The virtual arm started in a neutral position on the screen and targets appears around the screen in four fixed positions (Fig. 1c). Specifics for the control of the arm and the positioning of elements on the screen are the same as in our previous study [28]; however, the task was mirrored horizontally for left-side amputee subjects to align the movement of the virtual arm with the subject’s arm.

Subjects controlled the virtual arm to perform ballistic center-out reaches. With the cursor in the home circle (red hollow circle, Fig. 1c), a ball (grey filled circle) would appear above one of four targets. The ball would drop and align with the center of the target 0.5 s after the arm left the home circle. Subjects were instructed to reach towards the target, stopping when the ball reached the target, and with the cursor as close to the target as possible [31]. If the virtual limb did not come to a stop at the end of the trial (defined as both proximal and distal links moving slower than 45°/s), the ball was colored red. If the limb was successfully stopped but the cursor was not within the target, the ball remained grey. However, if the cursor was inside the target at the end of the trial (and the limb was sufficiently stilled), the ball was colored green to indicate a success.

### Familiarization

**Fig. 2:**
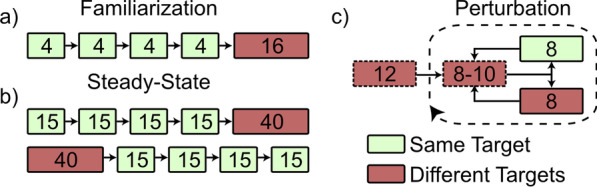
After one separate familiarization session, subjects completed the experimental protocol twice—once with and once without audio feedback. The order of the feedback and no feedback sessions was randomized across subjects. **a** The structured protocol for familiarization involved a total of 32 reaches: four sets of 4 reaches towards each target, and 16 reaches towards targets in balanced random order. **b** The steady-state block involved a total of 100 reaches: four sets of 15 reaches towards each target, and 40 reaches towards targets in balanced random order. The order of same- or different-target groupings was randomized across subjects and consistent between subject visits. **c** The Perturbation block started with 12 reaches towards targets in random order. After these baseline trials, subjects did cycles of 8–10 reaches towards targets in random order, followed by either 8 reaches towards the same target, or 8 reaches towards targets in balanced random order. The order of these cycles was randomized across subjects and consistent between subject visits. Reaches towards different targets with a dashed border indicate that balanced randomization was not enforced, and the number of reaches towards targets could differ from one another. Figure adapted from Earley et al. [28]

To learn to control the virtual arm, subjects began each visit with a familiarization session. During this session, subjects were provided time to understand the controller through unstructured exploration (subjects controlled the virtual limb with no visible targets), untimed target reaches (subjects reached towards targets but were given as much time as needed to complete each trial), and a structured protocol comprising 32 training ballistic center-out reaches. The first 16 trials had a specified reaching order (four sets of 4 reaches towards each target), and the second 16 trials had a balanced and randomized reaching order (4 reaches total towards each target) (Fig. 2a). No artificial feedback was provided during this session.

During the first visit, subjects only completed the Familiarization session. During the next two visits, subjects additionally completed a *feedback* protocol and a *no feedback* protocol in balanced randomized order. During the *feedback* protocol, subjects wore a pair of noise-canceling headphones (Bose QuietComfort 35 II) which played frequency-modulated tones determined by the speed of the wrist-driven distal link, where the pitch would increase by one octave for every multiple of 60°/s. During the *no feedback* protocol, subjects wore the noise-canceling headphones, but no sound was played.

### Steady-state block

To test trial-by-trial adaptation to self-generated errors, subjects completed two repetitions of 100 center-out reaches, each separated into one set of 60 and one set of 40 reaches (Fig. 2b). The order of these sets was randomized across subjects using balanced block randomization. Subjects were allowed a short break between sets.

During the set of 60 trials, subjects completed four sets of 10 reaches towards each target. During the set of 40 trials, subjects reached towards targets in a balanced and randomized order. After each set, expanding window optimization separated initial trials from steady-state trials for post-experiment analysis [32].

Two quantities were extracted from this trial-by-trial analysis. Adaptation rate was defined as the proportion of error from one trial that was corrected for in the following trial. Bias was defined as the amount of error which elicited no correction on average. It describes the intended reaching behavior, but is not necessarily the same as the average reaching error. This analysis was performed separately on the angular errors of both the elbow and the wrist, and was analyzed using a linear mixed effects model investigating main and interaction effects of the target set (*Same Targets (ST) or Different Targets (DT)*) and the feedback (*No Feedback (NFB) or Feedback (FB)*). A similar analysis was conducted on the magnitude of endpoint, elbow, and wrist errors. Subjects were coded as random variables, and *p*-values were adjusted using Holm-Bonferroni corrections.

A second stochastic signal processing approach was used to filter inherent motor control noise and provide unbiased estimates of true adaptation behavior [32–34]. This analysis provided outcomes for the internal model adaptation rate and the control noise; both were analyzed using the same linear mixed effects model as described above.

### Perturbation block

To test the speed of adaptation to external perturbations to the control system, subjects completed Perturbation blocks comprising 12 practice trials followed by 8 sets of perturbation trials. During each set, subjects started by making 8–10 unperturbed reaches towards random targets. The control system was then perturbed by doubling the EMG gain, which increased the speed of the distal link and made accurate and precise control more difficult. Subjects then made 8 reaches with the perturbed dynamics, either towards the *same target*, or towards *different targets*. Each category was tested in 4 sets of the perturbation trials (Fig. 2c). The order of these sets was determined randomly. The entire Perturbation block was repeated twice, yielding a total of 24 practice trials and 16 sets of perturbation trials—8 towards the *same target*, and 8 towards *different targets*.

Perturbation adaptation of the Euclidean distance between the cursor and the target was estimated using an exponential decay model which fit a gain ($\alpha$), decay rate ($\lambda$), and baseline error (${\varepsilon }_{\infty }$) to the perturbation trial data [35–37].

A hierarchical nonlinear mixed effects model described in a previous publication was intended to analyze data from the perturbation block [28]. However, this method was not viable due to the variability of reaches; thus, an exponential decay function was fit separately for each subject, combining all data for each condition, and the coefficients from these subject-based models were compared [38].

### Statistical analysis

Statistical analyses were performed using R-4.0.5. Linear mixed effects models investigated main and interaction effects for each analysis, and Holm-Bonferroni corrections were made for the number of terms in each model. Deidentified raw data and code for statistical analysis are publicly available on The Open Science Framework [39].

## Results

### Steady-state block

**Fig. 3:**
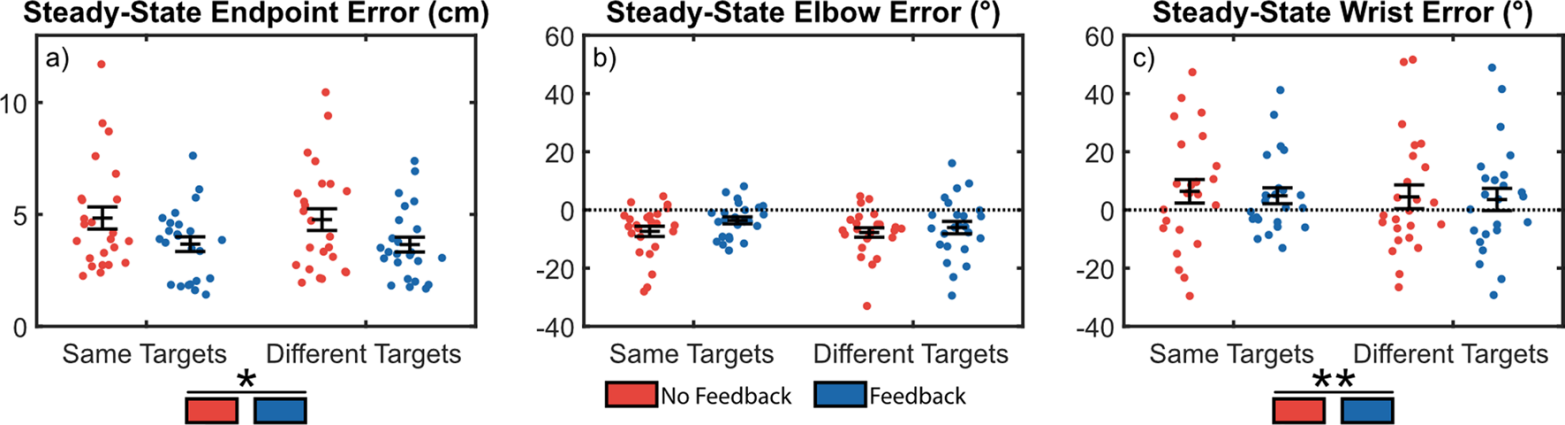
Endpoint and wrist angle errors were significantly reduced when wrist speed feedback was provided. Error bars indicate standard error of the mean. Errors are shown for reaches towards the same and different targets [1] for endpoint (**a**), position-controlled elbow (**b**), and myoelectric-controlled wrist (**c**). (*) indicates *p* < 0.05, (**) indicates *p* < 0.01

Steady-state reaches provide insight into how subjects coordinate positional- and myoelectric-controlled joints during reaching tasks after adapting to a control scheme, and may be used to quantify compensatory movements in one joint arising from errors or poor control in the other. Figure 3 shows the Euclidean endpoint errors (a) and joint angle errors (b, c).^1^

No significant interactions were found (*p*_*min*_ = 0.628), so interaction terms were removed and the models were rerun [40]. Endpoint errors were reduced with joint speed feedback available (FB mean ± SEM: 3.66 ± 0.65 cm) compared to when feedback was absent (NFB: 4.80 ± 0.97 cm, *p* = 0.047, Fig. 3a). Joint speed feedback also significantly reduced the magnitude of wrist angle errors (NFB: 15.42 ± 5.43°, FB: 11.94 ± 4.68°; *p* = 0.006, Fig. 3c), but did not significantly affect the magnitude of elbow angle errors (NFB: 8.42 ± 2.99°, FB: 7.42 ± 2.57°; *p* = 0.563, Fig. 3b). There were no significant differences when reaching towards the same or different targets for endpoint (ST: 4.26 ± 0.865, DT: 4.21 ± 0.85; *p* = 0.819), elbow (ST: 6.83 ± 2.59°, DT: 9.01 ± 2.92°; *p* = 0.563), or wrist (ST: 12.89 ± 4.88°, DT: 14.47 ± 5.33°; *p* = 0.588) errors.

**Fig. 4:**
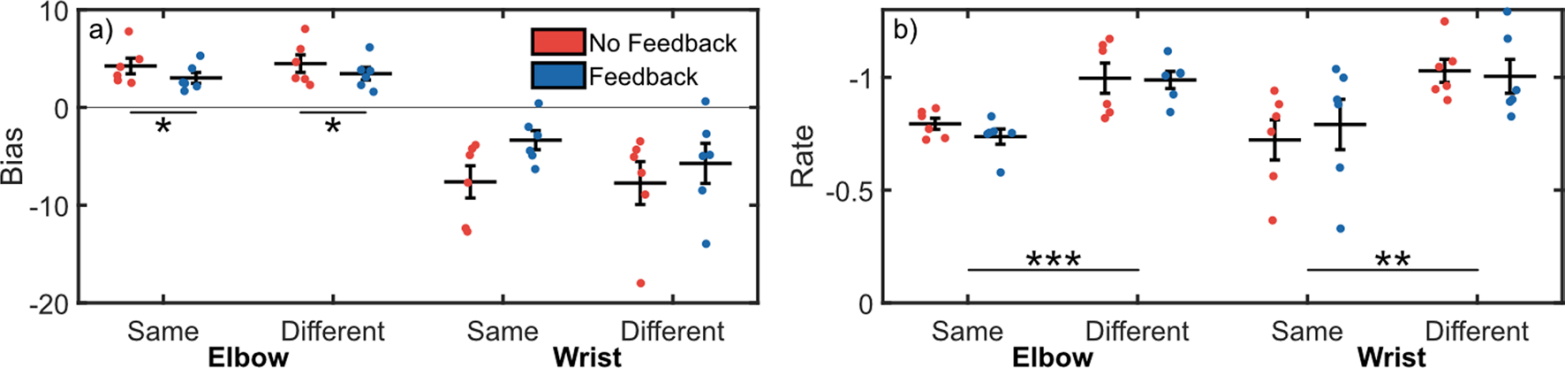
Trial-by-trial adaptation biases suggests the elbow overreaches to compensate for an underreaching wrist as shown by the opposite signs of elbow and wrist biases. However, no changes in trial-by-trial adaptation behavior were observed between feedback conditions. **a** Trial-by-trial adaptation bias. **b** Trial-by-trial adaptation rate. (*) indicated *p* < 0.05, (**) indicates *p* < 0.01, (***) indicates *p* < 0.001

We conducted an analysis of trial-by-trial adaptation to investigate differences in adaptation rates between *feedback* and *target* conditions, and to identify possible compensatory strategies in the reach biases. Our results showed no significant interactions between *feedback* and *target* for elbow bias or rate (*p*_*min*_ = 0.690), so the interaction terms were removed and the models rerun [40]. We found an improved adaptation rate during reaches towards different targets for the elbow (ST: − 0.77 ± 0.03, DT: − 0.99 ± 0.05; *p* < 0.001) and wrist (ST: − 0.76 ± 0.10, DT: − 1.02 ± 0.06; *p* = 0.010) (Fig. 4b), but no significant differences for the bias of the elbow (ST: 3.63 ± 0.70, DT: 3.97 ± 0.78; *p* = 0.414) and wrist (ST: − 5.48 ± 1.58, DT: − 6.73 ± 2.07; *p* = 0.358) (Fig. 4a).

No difference was observed between feedback conditions for wrist bias (NFB: − 7.68 ± 1.85, FB: − 4.54 ± 1.61; *p* = 0.060), but interestingly elbow bias was reduced (NFB: 4.40 ± 0.81, FB: 3.24 ± 0.58; *p* = 0.026). No differences were observed between feedback conditions for elbow (NFB: − 0.90 ± 0.07, FB: − 0.86 ± 0.06; *p* = 0.436) or wrist adaptation rates (NFB: − 0.88 ± 0.10, FB: − 0.90 ± 0.10; *p* = 0.794). Another interesting observation is that subjects attempted to under-reach with the wrist (demonstrated by the negative wrist bias) and overreach with the elbow (demonstrated by the positive elbow bias). Possible explanations for this reaching strategy are presented in "Discussion".

**Fig. 5:**
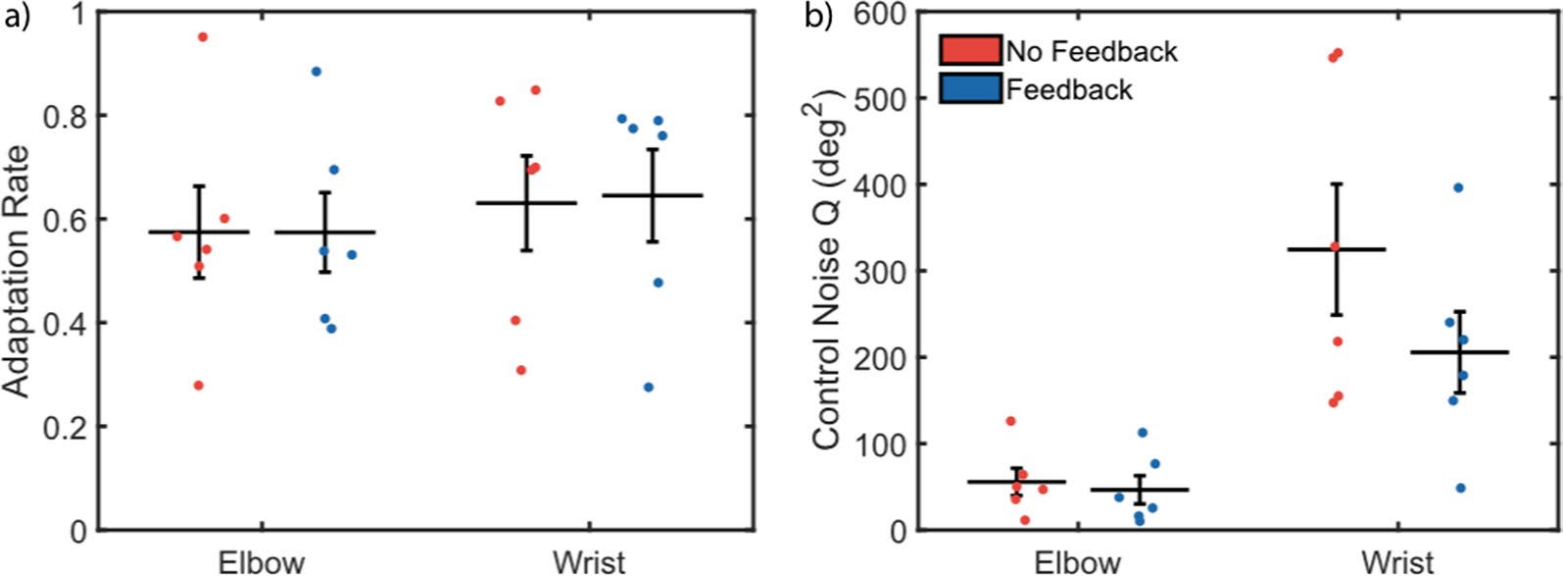
A secondary trial-by-trial analysis using stochastic signal processing approach found that **a** joint-speed feedback had no significant effect on adaptation rate for elbow or wrist movements, and **b** that although control noise was reduced with joint speed feedback for both elbow and wrist, these reductions were not significant

To supplement our traditional trial-by-trial analysis, we ran a secondary stochastic signal processing analysis. However, the data showed no significant change in adaptation rate for the elbow (NFB: 0.58 ± 0.09, FB: 0.57 ± 0.08; *p* = 0.996) or the wrist (NFB: 0.63 ± 0.09, FB: 0.65 ± 0.09; *p* = 0.887) (Fig. 5a). Analyzing the control noise (Q) similarly revealed no significant differences between feedback conditions for elbow (NFB: 55.71 ± 15.82 deg^2^, FB: 46.50 ± 16.38 deg^2^; *p* = 0.673) or wrist control noise (NFB: 324.58 ± 75.84 deg^2^, FB: 205.63 ± 46.98 deg^2^; *p* = 0.157) (Fig. 5b).

These results taken together suggest that joint speed feedback may improve the general accuracy of reaches (Fig. 3) and result in less compensatory movement bias of the intact joints (Fig. 4), which may translate to an improved confidence in the control of a myoelectric prosthesis. Further, the ratios of elbow-to-wrist biases may indicate a form to movement-based task optimization, such that improvements to myoelectric control can be detected in minor changes to bodily compensation within a constrained task. Reductions were also seen in elbow and wrist control noise (Fig. 5), though these differences were not statistically significant.

### Perturbation block

**Fig. 6:**
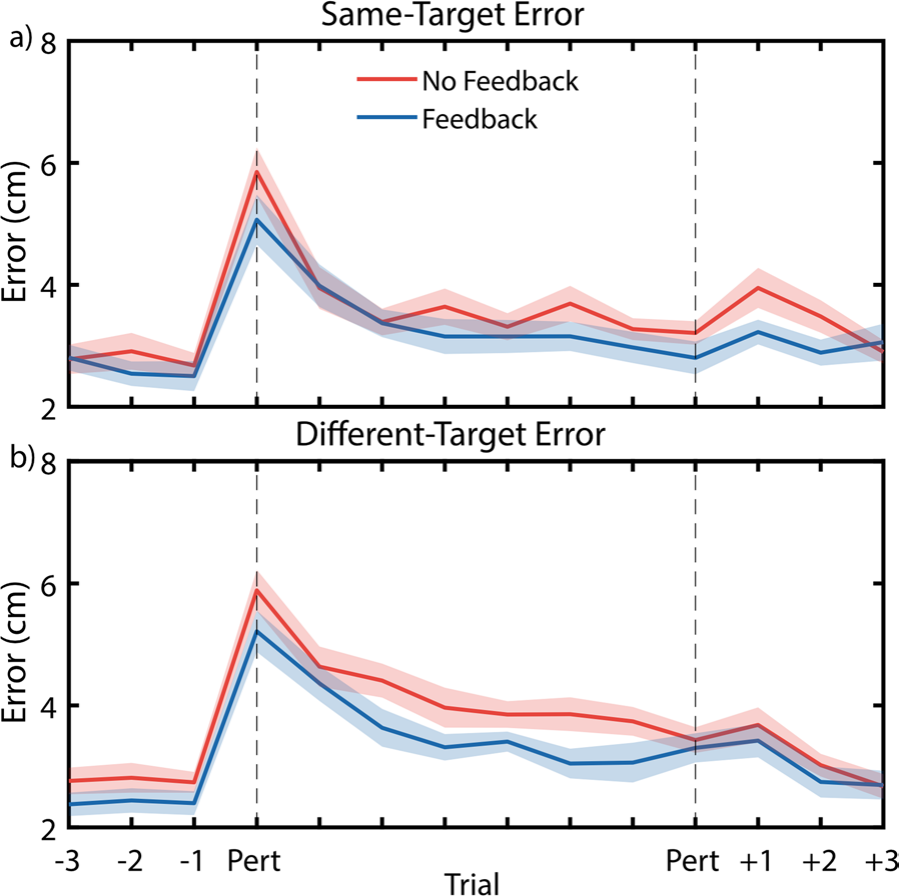
Average error traces during perturbation block show that feedback generally reduces errors prior to perturbation (as shown by the pre-perturbation trials and echoing Fig. 3a) but does not affect adaptation behaviors or reaching performance during perturbed reaches. Error traces are shown during perturbed reaches towards the same target (**a**) and different targets (**b**)

Perturbation trials test the ability for a person making reaches to adjust to suddenly changing task conditions, such as an abrupt change to the controller. Figure 6a, b shows the averaged subject responses to perturbation trials. The hierarchical nonlinear mixed effects model used in a previous study [28] was unable to run, likely due to insufficient and noisy data, thus individual exponential decay models were fit to each subject’s data for each condition, and the resulting coefficients were compared. However, no significant factors were uncovered from these statistical models (*p*_*min*_ = 0.533).

The magnitude of errors upon initial perturbation was not affected by feedback condition (*p* > 0.999) or target (*p* > 0.999). Final errors after adaptation to the perturbation were also not affected by feedback condition (*p* = 0.349) or target (*p* = 0.692). When final errors were subtracted from initial errors to determine the total improvement across the eight perturbation trials, this improvement was also found to not be affected by feedback condition (*p* = 0.563) or target (*p* = 0.759).

## Discussion

This study expanded upon our previous work by investigating transradial amputee performance during center-out reaching tasks. These tasks require coordination of elbow angle and wrist EMG to complete the reach. This paradigm differed from other similar studies into proprioceptive feedback by using a ballistic reach paradigm, which prevented subjects from incorporating feedback into their task performance and allowed us to investigate solely the impact of feedback on improvements to feedforward control [10, 13, 41, 42]. Our results provide some insight into how artificial joint speed feedback may be used to improve control of a myoelectric prosthesis. We found evidence that subjects were able to reduce their average reaching errors when provided audio feedback encoding the joint speed of a myoelectric limb (Fig. 3). We also found evidence suggesting the feedback may help prosthesis users reduce compensatory movement bias (Fig. 4a). However, no significant differences were found between feedback conditions for adaptation behavior after abrupt perturbations to the controller (Fig. 6).

In some aspects, our results agree with those from our previous study with non-amputee participants. Transradial amputee participants were able to complete ballistic center-out reaches requiring simultaneous control of positional- and myoelectric joints, in a manner similar to the how they may use their prosthesis in a home environment. Additionally, the same compensatory behavior was observed in both studies, where subjects would strategically under-reach with the wrist and compensate by overreaching with the elbow to minimize the distance to the target. This manner of compensatory movement is common for upper-limb prostheses (though normally demonstrated for the trunk and shoulder [43]), and aligns with the expected optimal reaching strategy to minimize endpoint error—given that only one limb configuration can reach each target, any wrist error can be optimally compensated with an opposing elbow adjustment of half the magnitude. It should be noted that, if reaching tasks required wrist flexion instead of wrist extension, the optimal reaching strategy to compensate for excessive flexion would be to overreach with the elbow. We also observe a more positive wrist error when reaching towards Target 3 (involving simultaneous extension of both the wrist and the elbow) than towards other targets, though interestingly the endpoint errors were lowest.^2^ This tendency may suggest that fine control of wrist extension is more difficult when coupled with simultaneous elbow extension. Alternatively, it may suggest that visual estimation of the requirement movement to achieve the target is more difficult. Interestingly, while we showed no impact of sensory feedback on the average errors in the previous study[28], amputee reaches in the present study demonstrated lower elbow bias, and a trend towards lower wrist bias, with feedback available (Fig. 4a).

The present study differs from the previous study with respect to steady-state errors; while no significant differences were observed in endpoint, elbow, or wrist errors for non-amputee reaches, transradial amputee endpoint and wrist angle errors were significantly improved with joint speed feedback. Furthermore, the stochastic analysis reveals an interesting difference between non-amputee and transradial amputee reaches: while elbow control noise is roughly equivalent between populations, the control noise of the myoelectric wrist can be more than twice as high for transradial amputees compared to non-amputees [28] (Fig. 5b).

However, where non-amputees demonstrated improved reaching errors after adapting to perturbations while reaching towards changing targets, transradial amputees showed no significant differences in perturbation adaptation behavior. One possible explanation for these inconclusive results stems from the heightened control noise. With myoelectric control noise for transradial amputees nearly double that of non-amputees, likely due to lack of or damage to proprioceptive organs, it is possible that this increased control noise led to increased internal model uncertainty, decreasing the capacity to adapt to perturbations. These trends may extend to adaptation behavior after control system perturbation. It should be noted that no individuals with congenital limb difference were included in this study; we expect control noise to be between those of transradial and non-amputees due to the more natural insertion of residual muscles, however this remains to be investigated.

The absence of incidental feedback may also contribute to the increased control noise observed in this study. When controlling a myoelectric prosthesis, users will generally rely on cues such as the sound and vibration of the hand as an indirect indicator of speed; the presence of these incidental cues in daily life may reduce control noise and consequently give rise to differences in adaptation behavior. In contrast, the auditory feedback modality used in this study provides a “best-case” scenario for low feedback uncertainty [27]; consequently, one might expect increased control noise when using vibrotactile or electrotactile feedback modalities with higher sensory uncertainty. This may even be the case when discrete vibrotactile feedback is fused with auditory feedback, as was seen in a recent study by Engels et al. [13].

Analyses in our current study were limited by the analysis methods available and the data collected for each. Our protocol required subjects to reach for several targets arranged throughout the reaching space, which ensured reaching performance was not localized to any one particular region. However, this also required splitting up reaches into smaller blocks of consistent reaches to prevent subject fatigue. As a result, adaptation models for self-generated errors were fit on relatively small amounts of data; this was especially the case for the stochastic signal processing analysis. Furthermore, this analysis requires a stationary target, thus reaches towards changing targets had to be omitted from this analysis. Analyzing self-generated error adaptation using two different methods allowed us to partially account for the limited data and build a fuller picture of adaptation behavior at steady-state.

The hierarchical model used our previous study requires sufficient data to fit all parameters across all included perturbation conditions [28]. Although the intent was to use the same model in this study, the smaller number of subjects prevented this model from converging. Furthermore, constraining the model parameters using insights from steady-state errors did not alleviate issues with model convergence [44]. In its place, we took an approach previously used in our pilot study [38]. In this approach, an individual exponential decay model is fit to each subject, for each condition. The coefficients from these models were then analyzed using a linear mixed effects model. To supplement this analysis, post-hoc comparisons were made on the initial and final errors achieved during perturbation trials. However, no significant differences were found during perturbation trials, whereas differences were found for non-amputee reaches.

The outcomes from the stochastic signal processing techniques also warrant additional attention. The non-improvement in the adaptation rate of the EMG-controlled wrist internal model is opposite of what is expected from reduced wrist noise. A possible explanation is that the high EMG control noise for transradial amputees, more than double than that of non-amputees at times, was more substantial than effects of volitional adaptation, which may have influenced the internal model adaptation rate as calculated using analytical methods [33]. It should be reiterated this analysis was conducted on relatively small amounts of data, which may disproportionately affect the variability or biases of calculated internal model adaptation rate.

The findings in this study corroborate those in a recent study on the clinical relevance of artificial feedback [19]. They conclude that the benefit of sensory feedback depends on the complexity of the task and the proficiency of the feedforward control. Our study involves a simple task—center-out reaching—made complicated by the control scheme. Our pilot study with trans-humeral amputees used a more difficult control scheme, and the high control noise made control (and adaptation) difficult [38] However, in our present experiment with trans-radial amputees, we show that improved feedback can reduce the control noise, thereby improving feedforward control [34]. This outcome suggests a need to test artificial sensory feedback systems with amputee patients of different levels to determine how beneficial feedback is to each population. Developing a more complete understanding of which factors determine the degree of benefit for prosthesis feedback can help researchers develop clinically impactful artificial sensory feedback which improves quality of life for people with amputations.

## Data Availability

All raw data and code for the experimental protocol, data analysis, and statistical analysis are freely available on the Open Science Framework [39].

## Abbreviations

DoF: Degree-of-freedom
EMG: Electromyography
NFB: *No Feedback*
FB: *Feedback*
ST: *Same Target*
DT: *Different Target*

## Footnotes

1 A supplemental variant of Fig. 3, which displays endpoint and joint angle errors separately for each reaching target, is provided alongside all other data and materials on the Open Science Framework [39].

2 This can be seen most clearly in the supplemental variant of Fig. 3, available on the Open Science Framework [39].
